# Supplementary material for: Characterization of Carbapenemase-Producing Klebsiella pneumoniae Isolates from Two Romanian Hospitals Co-Presenting Resistance and Heteroresistance to Colistin
Source: Antibiotics (Basel). 2022 Aug 30;11(9):1171. doi: 10.3390/antibiotics11091171 (PMC9495256; doi:10.3390/antibiotics11091171)
Supplement: Supplementary file 1 [file antibiotics-11-01171-s001.zip › Figure S1.pdf]

```
                      10         20         30         40         50         60         70         80         90        100        110        120        130
ompK35       MMKRNILAVV IPALLVAGAA NAAEIYNKNG NKLDFYGKMV GEHVWTTNGD TSSDDTTYAR IGLKGETQIN DQLIGYGQWE YNMDASNVEG SQTTKTRLAF AGLKAGEYGS FDYGRNYGAI YDVEAATDML
BC1_TM       .......... .......... .......... .......... .......... .......... .......... .......... .......... .......... .......... .......... ..........
BC2_BM       .......... .......... .......... .......... .......... .......... .......... .......... .......... .......... .......... .......... ..........
BC3_TM       .......... .......... .......... .......... .......... .......... .......... .......... .......... .......... .......... .......... ..........
BC4_BM       .......... .......... .......... .......... .......... .......... .......... .......... .......... .......... .......... .......... ..........
BC5_TM       .......... .......... .......... .......... .......... .......... .......... .......... .......... .......... .......... .......... ..........
BC6_BM       .......... .......... .......... .......... .......... .......... .......... .......... .......... .......... .......... .......... ..........
BC7_BM       .......... .......... .......... .......... .......... .......... .......... .......... .......... .......... .......... .......... ..........
BC8_BM       .......... .......... .......... .......... .......... .......... .......... .......... .......... .......... .......... .......... ..........
BC9_TM       .......... .......... .......... .......... .......... .......... .......... .......... .......... .......... .......... .......... ..........
BC10_TM      .......... .......... .......... .......... .......... .......... .......... .......... .......... .......... .......... .......... ..........
BC11_TM_B_hR .......... .......... .......... .......... .......... .......... .......... .......... .......... .......... .......... .......... ..........
BC12_TM_B_m  .......... .......... .......... .......... .......... .......... .......... .......... .......... .......... .......... .......... ..........
BC13_TM_C_hR .......... .......... .......... .......... .......... .......... .......... .......... .......... .......... .......... .......... ..........
BC14_TM_C_m  .......... .......... .......... .......... .......... .......... .......... .......... .......... .......... .......... .......... ..........

                     140        150        160        170        180        190        200        210        220        230        240        250        260
ompK35       VEWGGDGWNY TDNYMTGRTN GVATYRNSDF FGLVDGLSFA LQYQGKNDHD RAIRKQNGDG FSTAATYAFD NGIALSAGYS SSNRSVDQKA DGNGDKAEAW ATSAKYDANN IYAAVMYSQT YNMTPEEDNH
BC1_TM       .......... .......... .......... .......... .......... .......... .......... .......... .......... .......... .......... .......... ..........
BC2_BM       .......... .......... .......... .......... .......... .......... .......... .......... .......... .......... .......... .......... ..........
BC3_TM       .......... .......... .......... .......... .......... .......... .......... .......... .......... .......... .......... .......... ..........
BC4_BM       .......... .......... .......... .......... .......... .......... .......... .......... .......... .......... .......... .......... ..........
BC5_TM       .......... .......... .......... .......... .......... .......... .......... .......... .......... .......... .......... .......... ..........
BC6_BM       .......... .......... .......... .....XM... .......... .......... .......... .......... .......... .......... .......... .......... ..........
BC7_BM       .......... .......... .......... .......... .......... .......... .......... .......... .......... .......... .......... .......... ..........
BC8_BM       .......... .......... .......... .......... .......... .......... .......... .......... .......... .......... .......... .......... ..........
BC9_TM       .......... .......... .......... .......... .......... .......... .......... .......... .......... .......... .......... .......... ..........
BC10_TM      .......... .......... .......... .......... .......... .......... .......... .......... .......... .......... .......... .......... ..........
BC11_TM_B_hR .......... .......... .......... .......... .......... .......... .......... .......... .......... .......... .......... .......... ..........
BC12_TM_B_m  .......... .......... .......... .......... .......... .......... .......... .......... .......... .......... .......... .......... ..........
BC13_TM_C_hR .......... .......... .......... .......... .......... .......... .......... .......... .......... .......... .......... .......... ..........
BC14_TM_C_m  .......... .......... .......... .......... .......... .......... .......... .......... .......... .......... .......... .......... ..........

                     270        280        290        300        310        320        330        340        350        360
ompK35       FAGKTQNFEA VVQYQFDFGL RPSIGYVQTK GKDLQSRAGF SGGDADLVKY IEVGTWYYFN KNMNVYAAYK FNQLDDNDYT KAAGVATDDQ AAVGIVYQF*
BC1_TM       .......... .......... .......... .......... .......... .......... .......... .......... .......... .........*
BC2_BM       .......... .......... .......... .......... .......... .......... .......... .......... .......... .........*
BC3_TM       .......... .......... .......... .......... .......... .......... .......... .......... .......... .........*
BC4_BM       .......... .......... .......... .......... .......... .......... .......... .......... .......... .........*
BC5_TM       .......... .......... .......... .......... .......... .......... .......... .......... .......... .........*
BC6_BM       .......... .......... .......... .......... .......... .......... .......... .......... .......... .........*
BC7_BM       .......... .......... .......... .......... .......... .......... .......... .......... .......... .........*
BC8_BM       .......... .......... .......... .......... .......... .......... .......... .......... .......... .........*
BC9_TM       .......... .......... .......... .......... .......... .......... .......... .......... .......... .........*
BC10_TM      .......... .......... .......... .......... .......... .......... .......... .......... .......... .........*
BC11_TM_B_hR .......... .......... .......... .......... .......... .......... .......... .......... .......... .........*
BC12_TM_B_m  .......... .......... .......... .......... .......... .......... .......... .......... .......... .........*
BC13_TM_C_hR .......... .......... .......... .......... .......... .......... .......... .......... .......... .........*
BC14_TM_C_m  .......... .......... .......... .......... .......... .......... .......... .......... .......... .........*
```

```
                      10         20         30         40         50         60         70         80         90        100        110        120        130
ompK36        MKVKVLSLLV PALLVAGAAN AAEIYNKDGN KLDLYGKIDG LHYFSDDKNV DGDQTYMRLG VKGETQINDQ LTGYGQWEYN VQANNTESSS DQAWTRLAFA GLKFGDAGSF DYGRNYGVVY DVTSWTDVLP
BC1_TM        .......... .......... .......... .......... ........S. ........V. .......... .......... .......... .......... .......... .......... ..........
BC2_BM        .......... .......... .......... .......... ........S. ........V. .......... .......... .......... .......... .......... .......... ..........
BC3_TM        .......... .......... .......... .......... ........S. ........V. .......... .......... .......... .......... .......... .......... ..........
BC4_BM        .......... .......... .......... .......... ........S. ........V. .......... .......... .......... .......... .......... .......... ..........
BC5_TM        .......... .......... .......... .......... ........S. ........V. .......... .......... .......... .......... .......... .......... ..........
BC6_BM        .......... .......... .......... .......... ........S. ........V. .......... .......... .......... .......... .......... .......... ..........
BC7_BM        .......... .......... .......... .......... ........S. ........V. .......... .......... .......... .......... .......... .......... ..........
BC8_BM        .......... .......... .......... .......... ........S. ........V. .......... .......... .......... .......... .......... .......... ..........
BC9_TM        .......... .......... .......... .......... ........S. ........V. .......... .......... .......... .......... .......... .......... ..........
BC10_TM       .......... .......... .......... .......... ........S. ........V. .......... .......... .......... .......... .......... .......... ..........
BC11_TM_B_hR  .......... .......... .......... .......... ........S. ........V. .......... .......... .......... .......... .......... .......... ..........
BC12_TM_B_m   .......... .......... .......... .......... ........S. ........V. .......... .......... .......... .......... .......... .......... ..........
BC13_TM_C_hR  .......... .......... .......... .......... ........S. ........V. .......... .......... .......... .......... .......... .......... ..........
BC14_TM_C_m   .......... .......... .......... .......... ........S. ........V. .......... .......... .......... .......... .......... .......... ..........

                     140        150        160        170        180        190        200        210        220        230        240        250        260
ompK36        EFGGD--TYG SDNFLQSRAN GVATYRNSDF FGLVDGLNFA LQYQGKNGSV SGEGA---TN NGRGALKQNG DGFGTSVTYD IFDGISAGFA YANSKRTDDQ N-QLLLGEGD HAETYTGGLK YDANNIYLAT
BC1_TM        .....--... .......... .......... .......... .......... .....---.. K...WS.... ......L... .W........ .SH.....E. .SVPA..R.. N......... .........S
BC2_BM        .....GD... .......... .......... .......... .......... .....---.. ....WS.... ......L... .W........ .SH.....E. .SVPA..R.. N......... .........S
BC3_TM        .....--... .......... .......... .......... .......... .....LSP.. ...T...... ..Y...L... .Y........ .S....LG.. .SK.A..R.. N......... ..........
BC4_BM        .....GD... .......... .......... .......... .......... .....---.. ....WS.... ......L... .W........ .SH.....E. .SVPA..R.. N......... .........S
BC5_TM        .....GD... .......... .......... .......... .......... .....---.. ....WS.... ......L... .W........ .SH.....E. .SVPA..R.. N......... .........S
BC6_BM        .....--... .......... .......... .......... .........P .....LSP.. ...T...... ..Y...L... .Y........ .S....LG.. .SK.A..R.. N......... ..........
BC7_BM        .....--... .......... .......... .......... .......... ....T-SP.. .......... ......L... .Y........ .SH...NG.. .-R.DK.R.. N......... ..........
BC8_BM        .....--... .......... .......... .......... .......... .....LSP.. ...T...... ..Y...L... .Y........ .S....LG.. .SK.A..R.. N......... ..........
BC9_TM        .....GD... .......... .......... .......... .......... .....---.. ....WS.... ......L... .W........ .SH.....E. .SVPA..R.. N......... .........S
BC10_TM       .....GD... .......... .......... .......... .......... .....---.. ....WS.... ......L... .W........ .SH.....E. .SVPA..R.. N......... .........S
BC11_TM_B_hR  .....--... .......... .......... .......... .........P .....LSP.. ...T...... ..Y...L... .Y........ .S....LG.. .SK.A..R.. N......... ..........
BC12_TM_B_m   .....--... .......... .......... .......... .........P .....LSP.. ...T...... ..Y...L... .Y........ .S....LG.. .SK.A..R.. N......... ..........
BC13_TM_C_hR  .....--... .......... .......... .......... .........P .....LSP.. ...T...... ..Y...L... .Y........ .S....LG.. .SK.A..R.. N......... ..........
BC14_TM_C_m   .....--... .......... .......... .......... .........P .....LSP.. ...T...... ..Y...L... .Y........ .S....LG.. .SK.A..R.. N......... ..........

                     270        280        290        300        310        320        330        340        350        360        370
ompK36        QYTQTYNATR -------AGS LGFANKAQNF EVAAQYQFDF GLRPSVAYLQ SKGKDLN-GY GDQDILKYVD VGATYYFNKN MSTYVDYKIN LLDDNSFTRS AGISTDDVVA LGLVYQF*
BC1_TM        .......... -------... .......... ..V....... .......... ......ER.. .......... .......... .......... .........N .......... .......*
BC2_BM        .......... -------... .......... ..V....... .......... ......ER.. .......... .......... .......... .........N .......... .......*
BC3_TM        .......... -------... .......... ..V....... .......... ......E-.. .......... .......... .......... ........HN .......... .......*
BC4_BM        .......... -------... .......... ..V....... .......... ......ER.. .......... .......... .......... .........N .......... .......*
BC5_TM        .......... -------... .......... ..V....... .......... ......ER.. .......... .......... .......... .........N .......... .......*
BC6_BM        .......... -------... .......... ..V....... .......... ......E-.. .......... .......... .......... .........N .......... .......*
BC7_BM        .......... FSGNGESDSI S......... ..V....... .......... .....IE-.. ....L..... .......... .......... ...E.D...R .......... .......*
BC8_BM        .......... -------... .......... ..V....... .......... ......E-.. .......... .......... .......... ........HN .......... .......*
BC9_TM        .......... -------... .......... ..V....... .......... ......ER.. .......... .......... .......... .........N .......... .......*
BC10_TM       .......... -------... .......... ..V....... .......... ......ER.. .......... .......... .......... .........N .......... .......*
BC11_TM_B_hR  .......... -------... .......... ..V....... .......... ......E-.. .......... .......... .......... .........N .......... .......*
BC12_TM_B_m   .......... -------... .......... ..V....... .......... ......E-.. .......... .......... .......... .........N .......... .......*
BC13_TM_C_hR  .......... -------... .......... ..V....... .......... ......E-.. .......... .......... .......... .........N .......... .......*
BC14_TM_C_m   .......... -------... .......... ..V....... .......... ......E-.. .......... .......... .......... .........N .......... .......*
```

```
                       10         20         30         40         50         60         70         80         90        100        110        120        130
ompK37        MKRKVLALVI PALLAAGAAH AAEIYNKDGN KLDLYGKVDG LHYFSSDSKK DGDQTYLRFG FKGETQINDI LTGYGQWEYN VQANNTETSS DQAWTRLAFA GIKVGDYGSF DYGRNYGVLY DVEGWTDILP
BC1_TM        .......... .......... .......... .......... .......... .......... .........M .......... .......... .......... .......... .......... .......M..
BC2_BM        .......... .......... .......... .......... .......... .......... .........M .......... .......... .......... .......... .......... .......M..
BC3_TM        .......... .......... .......... .......... .......... .......... .........M .......... .......... .......... .......... .......... .......M..
BC4_BM        .......... .......... .......... .......... .......... .......... .........M .......... .......... .......... .......... .......... .......M..
BC5_TM        .......... .......... .......... .......... .......... .......... .........M .......... .......... .......... .......... .......... .......M..
BC6_BM        .......... .......... .......... .......... .......... .......... .........M .......... .......... .......... .......... .......... .......M..
BC7_BM        .......... .......... .......... .......... .......... .......... .........M .......... .......... .......... .......... .......... .......M..
BC8_BM        .......... .......... .......... .......... .......... .......... .........M .......... .......... .......... .......... .......... .......M..
BC9_TM        .......... .......... .......... .......... .......... .......... .........M .......... .......... .......... .......... .......... .......M..
BC10_TM       .......... .......... .......... .......... .......... .......... .........M .......... .......... .......... .......... .......... .......M..
BC11_TM_B_hR  .......... .......... .......... .......... .......... .......... .........M .......... .......... .......... .......... .......... .......M..
BC12_TM_B_m   .......... .......... .......... .......... .......... .......... .........M .......... .......... .......... .......... .......... .......M..
BC13_TM_C_hR  .......... .......... .......... .......... .......... .......... .........M .......... .......... .......... .......... .......... .......M..
BC14_TM_C_m   .......... .......... .......... .......... .......... .......... .........M .......... .......... .......... .......... .......... .......M..

                      140        150        160        170        180        190        200        210        220        230        240        250        260
ompK37        EFGGDSYTYA DNFMAGRANG VATYRNSDFF GLVEGLNFAL QYQGKNEGQN AQDINVGTNN RSSDSDVRFD NGDGFGLSTS YDFGMGISAA AAYTSSDRTN DQ----MTQT NARGDKAEAW TAGLKYDAND
BC1_TM        .......... .......... .......... .......... .......... .......... .......... .......... .......... .......... ..----.... .......... ..........
BC2_BM        .......... .......... .......... .......... .......... .......... .......... .......... .......... .......... ..----.... .......... ..........
BC3_TM        .......... .......... .......... .......... .......... .......... .......... .......... .......... .........G ..QHYTH.ER Y.K....D.. ..........
BC4_BM        .......... .......... .......... .......... .......... .......... .......... .......... .......... .......... ..----.... .......... ..........
BC5_TM        .......... .......... .......... .......... .......... .......... .......... .......... .......... .......... ..----.... .......... ..........
BC6_BM        .......... .......... .......... .......... .......... .......... .......... .......... .......... .......... ..----.... .......... ..........
BC7_BM        .......... .......... .......... .......... .......... .......... .......... .......... .......... .........G ..QHYTH.ER Y.K....D.. ..........
BC8_BM        .......... .......... .......... .......... .......... .......... .......... .......... .......... .........G ..QHYTH.ER Y.K....D.. ..........
BC9_TM        .......... .......... .......... .......... .......... .......... .......... .......... .......... .......... ..----.... .......... ..........
BC10_TM       .......... .......... .......... .......... .......... .......... .......... .......... .......... .......... ..----.... .......... ..........
BC11_TM_B_hR  .......... .......... .......... .......... .......... .......... .......... .......... .......... .......... ..----.... .......... ..........
BC12_TM_B_m   .......... .......... .......... .......... .......... .......... .......... .......... .......... .......... ..----.... .......... ..........
BC13_TM_C_hR  .......... .......... .......... .......... .......... .......... .......... .......... .......... .......... ..----.... .......... ..........
BC14_TM_C_m   .......... .......... .......... .......... .......... .......... .......... .......... .......... .......... ..----.... .......... ..........

                      270        280        290        300        310        320        330        340        350        360        370        380
ompK37        IYLATMYSET RNMTPYG--- ---NDGVANK TQNFEVTAQY QFDFGLRPAI SYLQSKGKDL YNNGRYADKD LVKYMDVGAT YYFNRNMSTY VDYKINLLDG NDKFYEDNGI STDNIVALGL VYQF*
BC1_TM        .......... .......--- ---....... .......... .......... .......... .......... .......... .......... .......... .......... .......... ....*
BC2_BM        .......... .......--- ---....... .......... .......... .......... .......... .......... .......... .......... .......... .......... ....*
BC3_TM        .......... .......STS STNGG.I... .......... .......... .......... .......... .......... .......... .......... .......... .......... ....*
BC4_BM        .......... .......--- ---....... .......... .......... .......... .......... .......... .......... .......... .......... .......... ....*
BC5_TM        .......... .......--- ---....... .......... .......... .......... .......... .......... .......... .......... .......... .......... ....*
BC6_BM        .......... .......--- ---....... .......... .......... .......... .......... .......... .......... .......... .......... .......... ....*
BC7_BM        .......... .......STS STNGG.I... .......... .......... .......... .......... .......... .......... .......... .......... .......... ....*
BC8_BM        .......... .......STS STNGG.I... .......... .......... .......... .......... .......... .......... .......... .......... .......... ....*
BC9_TM        .......... .......--- ---....... .......... .......... .......... .......... .......... .......... .......... .......... .......... ....*
BC10_TM       .......... .......--- ---....... .......... .......... .......... .......... .......... .......... .......... .......... .......... ....*
BC11_TM_B_hR  .......... .......--- ---....... .......... .......... .......... .......... .......... .......... .......... .......... .......... ....*
BC12_TM_B_m   .......... .......--- ---....... .......... .......... .......... .......... .......... .......... .......... .......... .......... ....*
BC13_TM_C_hR  .......... .......--- ---....... .......... .......... .......... .......... .......... .......... .......... .......... .......... ....*
BC14_TM_C_m   .......... .......--- ---....... .......... .......... .......... .......... .......... .......... .......... .......... .......... ....*
```

Figure S1. ClustalW alignment of *ompK35*, *ompK36*, and *ompK37* genes (BioEdit).
