# Supplementary material for: Characterization of Carbapenemase-Producing Klebsiella pneumoniae Isolates from Two Romanian Hospitals Co-Presenting Resistance and Heteroresistance to Colistin
Source: Antibiotics (Basel). 2022 Aug 30;11(9):1171. doi: 10.3390/antibiotics11091171 (PMC9495256; doi:10.3390/antibiotics11091171)
Supplement: Supplementary file 1 [file antibiotics-11-01171-s001.zip › Table S1.pdf]

Table S1. Mutations detected by ResFinder in *ompK36* and *ompK37* genes for all strains

Bacterial strain	Resistance to cephalosporins	Resistance to carbapenems
**BC1_TM**	*ompK36* (p.E232R), *ompK36* (p.L59V), *ompK36* (p.L191S), *ompK36* (p.F207W), *ompK36* (p.D224E), *ompK36* (p.T254S), *ompK36* (p.N49S), *ompK36* (p.L228V), *ompK36* (n304_None305insE), *ompK36* (p.Q227_None679del), *ompK36* (p.A190_None568del)	*ompK36* (p.N218H), *ompK36* (p.A217S), *ompK37* (p.I70M), *ompK37* (p.I128M)
**BC2_BM**	*ompK36* (p.L228V), *ompK36* (p.Q227_None679del), *ompK36* (p.E232R), *ompK36* (p.F207W), *ompK36* (p.T254S), *ompK36* (p.N49S), *ompK36* (n304_None305insE), *ompK36* (p.D224E), *ompK36* (p.L191S), *ompK36* (p.L59V), *ompK36* (p.A190_None568del)	*ompK36* (p.A217S), *ompK36* (p.N218H), *ompK37* (p.I70M), *ompK37* (p.I128M)
**BC3_TM**	*ompK36* (p.Q227_None679del), *ompK36* (p.D223G), *ompK36* (p.E232R), *ompK36* (p.F198Y), *ompK36* (p.N304E), *ompK36* (p.L59V), *ompK36* (p.T222L), *ompK36* (l228_None229insK), *ompK36* (p.N49S), *ompK36* (p.F207Y), *ompK36* (p.G189T)	*ompK37* (p.I128M), *ompK37* (p.I70M), *ompK36* (p.A217S), *ompK37* (m233_None234insQ), *ompK37* (p.N230G)
**BC4_BM**	*ompK36* (p.A190_None568del), *ompK36* (p.L59V), *ompK36* (p.D224E), *ompK36* (p.L228V), *ompK36* (p.F207W), *ompK36* (p.L191S), *ompK36* (n304_None305insE), *ompK36* (p.E232R),ompK36 (p.Q227_None679del), *ompK36* (p.T254S), *ompK36* (p.N49S)	*ompK37* (p.I128M), *ompK36* (p.N218H), *ompK36* (p.A217S), *ompK37* (p.I70M)
**BC5_TM**	*ompK36* (p.D224E), *ompK36* (p.L59V), *ompK36* (p.N49S), *ompK36* (p.T254S), *ompK36* (p.L228V), *ompK36* (p.E232R), *ompK36* (p.A190_None568del), *ompK36* (p.Q227_None679del), *ompK36* (p.F207W), *ompK36* (p.L191S), *ompK36* (n304_None305insE)	*ompK36* (p.N218H), *ompK37* (p.I128M), *ompK37* (p.I70M), *ompK36* (p.A217S)
**BC6_BM**	*ompK36* (p.N49S), *ompK36* (p.L59V), ompK36 (p.G189T), *ompK36* (p.F198Y), *ompK36* (p.F207Y), *ompK36* (p.T222L), *ompK36* (p.D223G), *ompK36* (p.Q227_None679del), *ompK36* (l228_None229insK), *ompK36* (p.E232R), *ompK36* (p.N304E),	*ompK37* (p.I128M), *ompK37* (p.I70M), *ompK36* (p.A217S)
**BC7_BM**	*ompK36* (p.T184P), *ompK36* (p.N49S), *ompK36* (p.L59V)	*ompK37* (p.N230G), *ompK37* (p.I70M), *ompK37* (p.I128M), *ompK37* (m233_None234insQ)
**BC8_BM**	*ompK36* (p.G189T), *ompK36* (l228_None229insK), *ompK36* (p.T222L), *ompK36* (p.N304E), *ompK36* (p.D223G), *ompK36* (p.Q227_None679del), *ompK36* (p.L59V), *ompK36* (p.F198Y), *ompK36* (p.F207Y), *ompK36* (p.E232R), *ompK36* (p.N49S)	*ompK37* (p.I128M), *ompK37* (p.N230G), *ompK37* (p.I70M), *ompK36* (p.A217S), *ompK37* (m233_None234insQ)

**BC9_TM**	*ompK36* (p.D224E), *ompK36* (p.E232R), *ompK36* (p.A190_None568del), *ompK36* (p.L191S), *ompK36* (p.Q227_None679del), *ompK36* (p.T254S), *ompK36* (p.L59V), *ompK36* (p.F207W), *ompK36* (p.L228V), *ompK36* (p.N49S), *ompK36* (n304_None305insE)	*ompK37* (p.I128M), *ompK36* (p.N218H), *ompK36* (p.A217S), *ompK37* (p.I70M)
**BC10_TM**	*ompK36* (p.D224E), *ompK36* (p.F207W), *ompK36* (p.L228V), *ompK36* (p.T254S), *ompK36* (p.A190_None568del), *ompK36* (n304_None305insE), *ompK36* (p.L59V), *ompK36* (p.N49S), *ompK36* (p.E232R), *ompK36* (p.Q227_None679del), *ompK36* (p.L191S)	*ompK37* (p.I70M), *ompK37* (p.I128M), *ompK36* (p.A217S), *ompK36* (p.N218H)
**BC11_TM_B_hR**	*ompK36* (p.N49S), *ompK36* (p.L59V), *ompK36* (p.G189T), *ompK36* (p.F198Y), *ompK36* (p.F207Y), *ompK36* (p.T222L), *ompK36* (p.D223G), *ompK36* (p.Q227_None679del), *ompK36* (l228_None229insK), *ompK36* (p.E232R), *ompK36* (p.N304E)	*ompK36* (p.A217S), *ompK37* (p.I70M), *ompK37* (p.I128M)
**BC12_TM_B_m**	*ompK36* (p.N49S), *ompK36* (p.L59V), *ompK36* (p.G189T), *ompK36* (p.F198Y), *ompK36* (p.F207Y), *ompK36* (p.T222L), *ompK36* (p.D223G), *ompK36* (p.Q227_None679del), *ompK36* (l228_None229insK), *ompK36* (p.E232R), *ompK36* (p.N304E),	*ompK37* (p.I128M), *ompK36* (p.A217S), *ompK37* (p.I70M)
**BC13_TM_C_hR**	*ompK36* (p.N49S), *ompK36* (p.L59V), *ompK36* (p.G189T), *ompK36* (p.F198Y), *ompK36* (p.F207Y), *ompK36* (p.T222L), *ompK36* (p.D223G), *ompK36* (p.Q227_None679del), *ompK36* (l228_None229insK), *ompK36* (p.E232R), *ompK36* (p.N304E),	*ompK36* (p.A217S), *ompK37* (p.I128M), *ompK37* (p.I70M)
**BC14_TM_C_m**	*ompK36* (p.N49S), *ompK36* (p.L59V), *ompK36* (p.G189T), *ompK36* (p.F198Y), *ompK36* (p.F207Y), *ompK36* (p.T222L), *ompK36* (p.D223G), *ompK36* (p.Q227_None679del), *ompK36* (l228_None229insK), *ompK36* (p.E232R), *ompK36* (p.N304E)	*ompK37* (p.I128M), *ompK36* (p.A217S), *ompK37* (p.I70M)

Legend. hR: heteroresistant; m: mutant.
